# Update on the Risk Factors for Thyroid Dysfunction in Pregnancy

**DOI:** 10.3390/biomedicines14030564

**Published:** 2026-03-01

**Authors:** Federica De Luca, Roberto Negro, Stella Bernardi

**Affiliations:** 1 Department of Medical Surgical and Health Sciences, University of Trieste, 34127 Trieste, Italy; federica.deluca@studenti.units.it; 2 Department of Experimental Medicine, University of Salento, 73100 Lecce, Italy; roberto.negro@unisalento.it

**Keywords:** thyroid, pregnancy, risk factors, screening

## Abstract

Thyroid disorders are common in pregnancy and if left untreated, they can have serious consequences. There is an ongoing debate as to whether thyroid screening in pregnancy should be universal. Current guidelines recommend a risk-based thyroid screening, which is a health strategy offering TSH measurement to higher-risk women only. Given the key role of risk stratification, in this narrative review, we aimed to (i) describe the established risk factors for thyroid disease in pregnancy; (ii) provide an update on the emerging risk factors; (iii) examine their overall potential utility. For this purpose, a comprehensive literature search was conducted across the PubMed, Scopus, and Web of Science databases, employing a combination of MeSH terms and relevant keywords, including ‘thyroid dysfunction’, ‘pregnancy’, ‘risk factors’, ‘screening’. In conclusion, the current literature confirms that thyroid autoimmunity and moderate-to-severe iodine deficiency remain the most consistent and reproducible predictors of thyroid dysfunction.

## 1. Introduction

Thyroid disorders are common endocrine abnormalities that can develop during pregnancy. They include not only overt but also—and most importantly—subclinical forms of hypothyroidism and hyperthyroidism. Although their prevalence varies across studies and populations, overt hypothyroidism is estimated to occur in 2–3% of pregnancies, while overt hyperthyroidism in 0.1–0.4%, and subclinical hypothyroidism in 4–15% [1,2,3]. The prevalence of thyroid autoimmunity varies between 2 and 17% [1,3,4,5]. Given the importance of thyroid hormones for maternal and child health, early identification and management of thyroid disorders during pregnancy is crucial for both [6].

Otherwise, untreated or inadequately addressed overt hypothyroidism is associated with an increased risk of spontaneous miscarriage, perinatal death, pre-eclampsia, pregnancy-induced hypertension, preterm birth, low birth weight, and postpartum hemorrhage [7,8,9,10,11]. In addition, due to the fact that thyroid hormones are essential for normal brain development [12], overt hypothyroidism can impair the neurocognitive and neuropsychological development of the child [13]. For instance, Haddow et al. showed that undiagnosed and untreated hypothyroidism adversely affected fetal neurocognitive development in pregnant women, as their children showed significantly lower neuropsychological test scores as compared to those of control women and those of hypothyroid women that received levothyroxine during pregnancy [13]. Also, overt hyperthyroidism, commonly due to Graves’ disease, is associated with an increased risk of pre-eclampsia, preterm birth, fetal growth restriction, and maternal heart failure [14].

In light of the above, there is an ongoing debate as to whether thyroid screening in pregnancy should be universal. The implementation of universal screening can be suggested on the basis that overt hypothyroidism is a prevalent condition, asymptomatic in most cases, has serious consequences if untreated, is detectable with blood tests, and easily managed with levothyroxine. In this context, it has been demonstrated that universal screening of pregnant women in the first trimester is cost-effective, not only compared with no screening but also compared with screening of high-risk women [15]. However, based on the fact that universal thyroid screening in pregnancy would reveal mostly cases of subclinical thyroid dysfunction, where the benefit of treatment has not yet been proven conclusively [16,17,18,19,20,21,22,23,24], current guidelines recommend a risk-based thyroid screening in pregnant women [1,2,3].

Risk-based or targeted thyroid screening is a health strategy offering TSH measurement to higher-risk women only. Given the key role of risk stratification, in this narrative review, we aimed to (i) describe the established risk factors for thyroid disease in pregnancy; (ii) provide an update on the emerging risk factors for thyroid disease in pregnancy; (iii) examine their overall potential utility.

For this purpose, a comprehensive literature search was conducted across the PubMed, Scopus, and Web of Science databases, covering articles published up to December 2025. The search strategy employed a combination of MeSH terms and relevant keywords, including ‘thyroid dysfunction’, ‘pregnancy’, ‘risk factors’, ‘screening’. We prioritized recent high-quality studies, including systematic reviews, meta-analyses, and clinical guidelines published within the last 10 years, although landmark historical studies were also included where appropriate. Selection criteria focused on English-language original research and reviews that provided mechanistic insights or clinical evidence regarding established and emerging risk factors for gestational thyroid disease.

## 2. Established Risk Factors for Thyroid Disease in Pregnancy

### 2.1. Overview on the Risk Factors for Thyroid Disease in Pregnancy

Current guidelines recommend risk-based thyroid screening in pregnancy, which means that TSH should be measured only in pregnant women with risk factors for thyroid dysfunction, ideally at first contact with a healthcare professional by the middle of the first trimester [1,2,3]. According to current clinical guidelines (i.e., ATA guidelines [1]), several established factors identify women at high risk for thyroid dysfunction during pregnancy. These include an age over 30 years, a body mass index (BMI) ≥ 40 kg/m^2^, a personal history or clinical signs and symptoms of thyroid dysfunction, the presence of a goiter, and positivity for thyroid antibodies. The latter is of particular clinical relevance, as it was clearly shown that women with asymptomatic autoimmune thyroid disease who are euthyroid in early pregnancy carry a significant risk of developing hypothyroidism progressively during gestation [25]. Similarly, type 1 diabetes mellitus (T1DM) or other autoimmune diseases are major risk factors; indeed, overt hypothyroidism has been reported in 16% of women with T1DM [26] and 11% of those with systemic lupus erythematosus [27]. Furthermore, a family history of thyroid autoimmunity or dysfunction significantly increases the risk. Environmental and medical history also play a crucial role, specifically residing in areas with moderate-to-severe iodine deficiency, prior thyroid surgery, or the use of medications such as amiodarone and lithium, including recent exposure to iodinated contrast media. A history of head or neck radiation is also a major determinant; notably, it was shown that eight years after head and neck irradiation, up to 67% of women developed hypothyroidism [28]. Finally, obstetric and reproductive history are key indicators, including infertility, multiparity (having had two or more prior pregnancies), and a history of preterm delivery or pregnancy loss; notably, the latter is a significant marker as autoimmune thyroid disease and overt or subclinical thyroid dysfunction have been associated with an increased risk of pregnancy loss [29,30,31,32].

### 2.2. Thyroid Autoimmunity

Veltri and Poppe have argued that thyroid function variability during pregnancy is primarily driven by genetic factors and thyroid autoimmunity [33]. On the one hand, genetic factors account for 40–65% of the inter-individual variation in serum thyroid hormones, reflecting a unique hypothalamic-pituitary-thyroid axis set point for each woman [34]. On the other, it has been largely demonstrated that thyroid autoimmunity, which is present in up to 17% of pregnant women [1], can significantly affect thyroid homeostasis. Autoimmune thyroid disease induces an inadequate thyroid capacity for the increased hormone production imposed by pregnancy, due to chorionic gonadotropin (hCG) and the increase in thyroxine-binding gobulin (TBG). For instance, more than 40% of women with thyroid autoimmunity present with fT4 levels in the range of hypothyroidsm in late pregnancy [25].

Recent evidence confirms this association. A meta-analysis of 51,138 pregnant women showed that TSH levels increase with isolated thyroid peroxidase antibody (TPOAb) or thyroglobulin antibody (TgAb) positivity, which is significantly amplified in women positive for both [35]. Furthermore, an individual-participant data meta-analysis of 65,559 women has identified thyroid autoimmunity as a powerful predictor of dysfunction: compared to antibody-negative women, those with dual TPOAb/TgAb positivity showed a dramatically higher incidence of overt (7.0% vs. 0.1%; OR 24.2) and subclinical hypothyroidism (20.0% vs. 2.2%; OR 8.42) [36]. Isolated positivity for either antibody also conferred an intermediate but significantly elevated risk. These findings underscore the clinical importance of testing for both markers; specifically, incorporating TPOAb and TgAb status into risk-based screening allows for a more accurate identification of women at risk of gestational thyroid failure [37]. Kotval has recently put forward considering the incorporation of both TPOAb and TgAb assessment into risk-based screening strategies [37], which should be performed in the first trimester [38].

### 2.3. Iodine Deficiency

**Figure 1:**
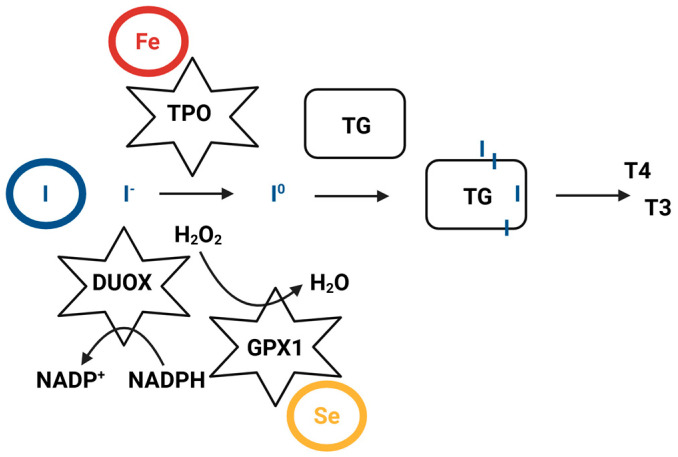
Key elements implicated in thyroid hormone biosynthesis. DUOX, dual oxidase; Fe, iron; GPX1, gluthatione peroxidase 1; I, iodine; I^-^, iodide; I^0^, active iodine; NADP, oxidized form of nicotinamide adenosine dinucleotide phosphate; NADPH, reduced nicotinamide adenosine dinucleotide phosphate; T3, triiodothyronine; T4, thyroxine; TG, thyroglobulin; TPO, thyroid peroxidase; Se, selenium.

Iodine is a trace element essential for thyroid hormone synthesis, which begins with iodine incorporation into tyrosine residues of thyroglobulin (Figure 1).

Iodine requirements increase in pregnancy due to heightened hormone production, elevated renal iodine clearance, and the active transfer of iodine to the fetus. These physiological changes raise the recommended daily intake from 150 μg/day to 220–250 μg/day in pregnant women [6]. Severe iodine deficiency (intake < 50 μg/day and urinary iodine < 20 μg/L) is the most critical cause of maternal hypothyroidism, impairing thyroxine synthesis in both mother and fetus, which is fundamental for fetal neurogenesis [39]. Historical evidence has shown that severe deficiency led to endemic cretinism, characterized by severe intellectual disability, deaf-mutism and stunted growth [40,41]. To address this, the term “iodine deficiency disorders” (IDDs) was introduced in 1983 to encompass the broad spectrum of iodine-related conditions, including miscarriages and congenital abnormalities [42]. Iodine supplementation in severely iodine-deficient regions has been shown to decrease the rate of cretinism [43], improve birth outcomes [44], and increase offspring intelligence quotient (IQ) [45,46]. Consequently, mandatory salt iodization has been implemented in over 120 countries, and today, approximately 88% of the global population has access to iodized salt [47]. Of note, a recent work by Wang et al. has shown that moderate-to-severe iodine deficiency (urinary iodine < 100 μg/L) in the 1st trimester was associated with a significantly higher risk of subclinical hypothyroidism during the 2nd or 3rd trimesters (OR = 2.47; 95%CI = 1.22–5.71) among euthyroid pregnant women without thyroid autoimmunity [48].

While the benefits of correcting severe iodine deficiency are undisputed, the impact of mild-to-moderate iodine deficiency in pregnancy (urinary iodine 50–150 μg/L) remains complex [3]. In this setting, maternal thyroid hormone levels often remain within reference ranges [49]. Large-scale birth cohorts, including the Generation R, INMA, and ALSPAC projects [50,51,52], as well as a Cochrane meta-analysis [53], did not show consistent associations between mild-to-moderate deficiency and immediate obstetric outcomes such as birth weight or fetal growth. On the other hand, long-term neurodevelopmental data offer a different perspective. The ALSPAC project and studies by Hynes et al. showed that children of mothers with suboptimal iodine status (iodine-to-creatinine ratio of 50–150 μg/g) faced an increased risk of lower scores in verbal IQ, reading comprehension and poorer educational outcomes at ages 8–9 [54]. Furthermore, while some studies found a link between low iodine and attention deficit hyperactivity disorder (ADHD) symptoms [55], recent collaborative analyses did not confirm this link [56], arguing that inconsistent results may be due to the fact that urinary iodine concentration is a proxy for individual iodine status and the timing of supplementation is crucial. Evidence from Levie et al. suggests that the neurodevelopmental benefits of supplementation are primarily observed when initiated before the 14th week of gestation [57]. Therefore, achieving optimal iodine intake is essential in early pregnancy [1,2], or ideally, during the preconception period. In this context, the American Thyroid Association recommends a daily supplement of 150 µg for women who are pregnant, lactating, or planning pregnancy [1].

## 3. Emerging Risk Factors for Thyroid Disease in Pregnancy

Data from the literature suggest that there are many other potential variables that may contribute to thyroid (dys)function in pregnant women, which are not included in current guidelines. These are environmental factors, including iodine excess, iron deficiency, and exposure to endocrine disruptors; constitutive factors, such as obesity (BMI > 30 Kg/m^2^) and ethnicity; as well as pregnancy-related factors, such as human chorionic gonadotropin (hCG) and soluble fms-like tyrosine kinase-1 (sFlt-1)/placental growth factor (PlGF) ratio.

### 3.1. Iodine Excess

Recent studies demonstrate that not only iodine deficiency but also iodine excess is a risk factor for thyroid disease in pregnancy. The primary mechanism by which iodine excess leads to thyroid dysfunction is the Wolff-Chaikoff effect, an autoregulatory process where high concentrations of intrathyroidal inorganic iodide acutely inhibit thyroid peroxidase, the organification of iodine, and thyroid hormone synthesis [58]. While healthy individuals typically ‘escape’ from this effect within a few days through the downregulation of the sodium-iodide symporter (NIS), a failure in this escape mechanism can lead to iodine-induced hypothyroidism [58]. Pregnant women may be particularly vulnerable to this phenomenon as they must increase iodine intake to meet physiological demands [59]. In addition, the fetal thyroid gland, which cannot efficiently escape from the Wolff-Chaikoff effect until late gestation (around the 36th week) is highly susceptible to the inhibition of hormone production, increasing the risk of fetal hypothyroidism and goiter [60]. Furthermore, excessive iodine intake may enhance the immunogenicity of thyroglobulin, potentially triggering or exacerbating autoimmune thyroiditis in genetically susceptible women [59,61].

Epidemiological studies demonstrate a U-shaped relationship between maternal urinary iodine concentration and thyroid dysfunction [62]. In particular, evidence suggests that women with more-than-adequate or excessive iodine intake (urinary iodine ≥ 250 μg/L) face a significantly higher risk of subclinical hypothyroidism—with odds ratios reaching 1.72 and 2.17, respectively [62]—which may also negatively impact infantile neurodevelopment [63]. Interestingly, a recent Danish nationwide study observed a steady increase in maternal hypothyroidism following mandatory iodine fortification, with a higher prevalence in regions with higher iodine intake [64]. Whether strictly related to iodine fortification or not, these results underline the importance of tailored global policies that account for regional differences in iodine status to prevent the adverse effects of both deficiency and excess. In this context, regarding the recommended daily supplement of 150 µg for pregnant, lactating, or preconception women, the iodine doses found in standard prenatal vitamins seem unlikely to induce the aforementioned adverse effects [3].

### 3.2. Iron Deficiency

Iron plays a crucial role in thyroid hormone biosynthesis as a core component of thyroid peroxidase (TPO), which is a heme-dependent enzyme (Figure 1). Consequently, iron deficiency (ID) impairs the heme-dependent TPO activity, leading to a reduction in circulating T3 and T4 levels [65]. Consistent with this, it has been shown that ID reduces the effectiveness of iodine prophylaxis in areas with endemic goiter, while iron replacement improves it [66].

ID arises when physiological requirements cannot be met by iron intake through diet or absorption and it can be diagnosed by serum ferritin levels < 15μg/L [67,68]. Pregnant women are particularly vulnerable to this condition, due to the expansion of red blood cell mass and the increased iron demand to support fetal growth [67]. Specifically, total iron requirements during pregnancy are estimated at approximately 1 g, coming from 230 mg for maternal basal losses, 360 mg for feto-placental development, and 450 mg for red blood cell expansion [67]. Consequently ID is extremely frequent among pregnant women, particularly in the second half of the gestation, with a prevalence ranging from 24% to 44% of women in industrialized countries [69]. This was confirmed in a recent population-based study by Moreno-Reyes et al. [68], showing that the frequency of serum ferritin < 15 μg/L rose from 6.2% in the first trimester to 39.6% in the third. Of note, ID can be even more prevalent in other regions of the world; for instance, Gupta et al. recently demonstrated a 68% prevalence among pregnant women [70].

Several authors have reported that ID predicts poor maternal thyroid status during pregnancy in borderline areas of iodine deficiency. In particular, Zimmerman et al. demonstrated that iron deficiency predicted both higher TSH and lower total T4 concentration in an area with borderline iodine intake [71]. Likewise, Moreno-Reyes et al. observed that the frequency of fT4 below the 10th percentile in the third trimester was significantly higher in women with negative body iron stores (24% vs. 14%) and borderline iodine deficiency. In the first trimester, ferritin and body iron stores were significant predictors of fT4 and T4, while hemoglobin was a significant predictor of fT4 in both trimesters and of T4 in the third trimester [68]. In addition, Veltri et al. showed a higher prevalence of thyroid autoimmunity in women with ID during the first trimester [72]. To further clarify the relationship between iron and thyroid function, Yu et al. studied an iodine-adequate area in China, demonstrating that ID was associated with isolated hypothyroxinemia in both pregnant and non-pregnant women, independently of iodine status and thyroid autoimmunity [73]. Finally, a recent meta-analysis, including reproductive-age and pregnant women from Belgium, Turkey, and China, confirmed that pregnant women with ID have significantly increased serum TSH levels and decreased fT4 levels. In this work, the risk of both overt (OR: 1.60; 95%CI: 1.17, 2.19) and subclinical hypothyroidism (OR: 1.37; 95%CI: 1.13, 1.66) were significantly increased in the presence of ID [74].

### 3.3. Endocrine Disruptor Chemicals

**Figure 2:**
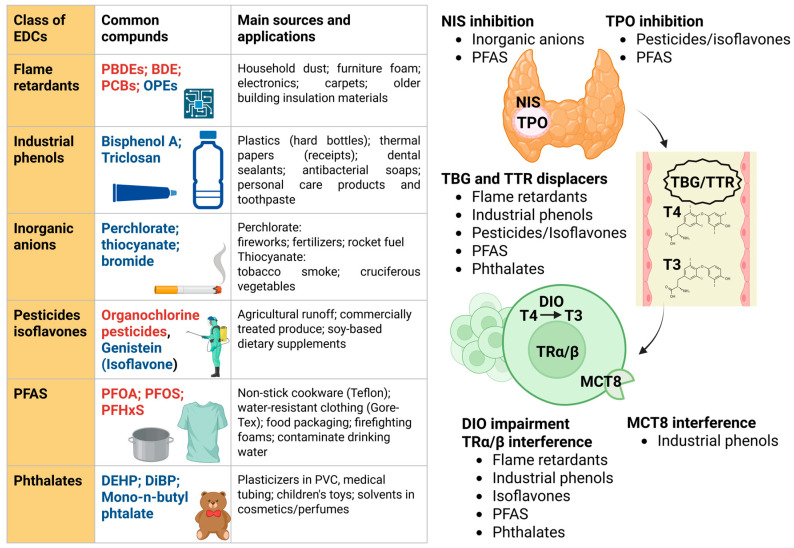
Classification, sources, and thyroid-disrupting mechanisms of Endocrine-Disrupting Chemicals (EDCs). The left panel categorizes the major classes of EDCs (flame retardants, industrial phenols, inorganic anions, pesticides/isoflavones, PFAS, and phthalates) alongside their common chemical compounds and primary environmental or household sources of exposure. Of note, Persistent Organic Pollutants (POPs), such as PFAS, PBDEs, PCBs, and organochlorine pesticides, which are in red ink, are characterized by long half-lives and bioaccumulation in adipose tissue. Conversely, non-persistent chemicals, including phthalates, phenols (BPA), isoflavones, inorganic anions, and modern organophosphate esters (OPEs), which are in blue ink, are rapidly metabolized but pose a continuous risk due to ubiquitous daily exposure. The right panel illustrates the pathophysiological targets of these substances within the thyroid system. Intrathayroidal interference: Inhibition of the sodium-iodide symporter (NIS), reducing iodine uptake, and inhibition of thyroid peroxidase (TPO), impairing thyroid hormone synthesis. Transport disruption: Displacement of thyroxine (T4) and triiodothyronine (T3) from plasma-binding proteins, specifically Thyroxine-Binding Globulin (TBG) and Transthyretin (TTR). Peripheral and cellular interference: interference with the Monocarboxylate Transporter 8 (MCT8), which mediates cellular hormone uptake; disruption of deiodinase (DIO) activity affecting the peripheral conversion of T4 to T3; impairment of thyroid hormone receptors (TH receptors).

In 2002 the World Health Organization defined endocrine disruptor chemicals (EDCs) as exogenous substances or mixtures interfering with the normal functioning of the endocrine system, leading to detrimental health effects in the exposed individuals, their offspring, or specific subpopulations [75]. Figure 2 shows the classification, sources, and thyroid-disrupting mechanisms of Endocrine-Disrupting Chemicals (EDCs). Briefly, sources of EDCs include drinking water, air pollution, pesticides, agricultural chemicals, flame retardants, cleaning agents, personal care products, food additives and packaging, coatings, solvents, as well as medical products and equipment [76]. The thyroid and the thyroid hormone singnaling are affected by many classes of EDCs [75] in almost every step. They include thyroid hormone synthesis, which begins with iodide uptake via the sodium-iodide symporter (NIS), and oxidation by thyroid peroxidase (TPO). Once in circulation, hormones are bound to plasma carriers like thyroxine-binding globulin (TBG) and transthyretin (TTR), which increase significantly during pregnancy. Cellular uptake is then mediated by membrane transporters such as monocarboxylate transporter 8 (MCT8), while intracellular availability is refined by deiodinases (DIO1-3). Finally, signaling is mediated by nuclear receptors (TRα and TRβ).

All EDCs actions on thyroid hormone signaling have been recently reviewed by Kohrle et al. [75] and Pearce et al. [76]. Based on current evidence, it seems that perchlorate, thiocyanate and per- and poly-fluorolakyl substances (PFAS) inhibit the NIS, reducing iodine uptake. Pesticides, isoflavones, and PFAS inhibit TPO activity. In addition, bisphenol A (BPA) and tetrabromobisphenol A, isoflavones, mono-n-butyl phthalate, bromodiphenyl ethers (BDEs), polybrominated diphenyl ethers (PBDEs), polychlorinated biphenyls (PCBs), PFAS, and perfluorooctanesulfonic acid (PFOS) can displace thyroid hormones from the binding globulins TBG and TTR. BPA interferes with MCT8 and the transportation of thyroid hormones into cells. BDEs, BPA, triclosan, butyl paraben, PBDEs, PCBs, isoflavonoids, PFAS, and phthalates interfere with deiodinases. Chemicals such as BPA, PBDEs, PCBs, PFAS, and triclosan, which have structural homology to thyroid hormones, can bind to and act as agonists or antagonists at thyroid hormone receptors. In addition, EDCs may decrease the half-life of thyroxine (T4) by inducing activity of hepatic uridine diphosphate glucuronosyltransferrases or sulfotransferases, which metabolize thyroid hormones. Having said that, it has been argued that we are not exposed to one single thyroid disruptor chemical, but to their mixture, and this constellation of chemical-disrupting agents creates major, mainly unsolved, challenges in experimental, empirical, epidemiological, and clinical hazard identification and potential risk assessment for EDCs [75].

Pregnant women are particularly vulnerable to the effects of thyroidal EDCs in case of iodine deficiency or the presence of autoimmunity. For example, in pregnant women perchlorate and thiocyanate, which are NIS inhibitors, were associated with high TSH and low fT4 levels [77]. In a recent study, it was also shown that several long-chain PFAS were associated with disrupted thyroid homeostasis in Chinese pregnant women, especially among TPO antibody-positive women [78]. In another case-control study in Chinese women, urinary monoethyl phthalate (MEP) levels were observably higher in women with subclinical hypothyroidism than in those without. In this study, mono-(2-ethyl-5-carboxypentyl) phthalate (MECPP), MEP, mono-(2-ethyl-5-oxohexyl) phthalate (MEOHP), and di-(2-ethylhexyl) phthalate (ΣDEHP) were significantly associated with a higher risk of SCH during early pregnancy (adjusted odds ratios = 1.89, 1.42, 1.81, and 1.92, respectively) [79].These effects can be amplified by genetic predisposition. For instance, pregnant women carriers of deiodinase1 rs2235544 genetic variant exhibited a different response to chlorinated EDCs in terms of thyroid hormone levels [80]. Otherwise, it is important to note that the clinical impact of EDCs depends significantly on the type of chemical but also on the timing of exposure during gestation. In early pregnancy (first trimester), EDCs primarily affect maternal thyroid hormone availability [81,82], which is crucial for fetal neurodevelopment [83]. In addition, in early pregnancy, exposure to EDCs has been associated with implantation failure and miscarriage risk. During mid-pregnancy, exposure to EDCs has been associated with impaired placental angiogenesis, growth restriction, and neurodevelopmental disorders, while in late pregnancy, it has been associated with pre-eclampsia risk, impaired glucose metabolism, and preterm delivery [84,85]. It should be noted that as the fetal thyroid begins to function, exposure to mixtures of EDCs can directly disrupt fetal thyroid homeostasis as well [86].

### 3.4. Obesity

Obesity is an increasingly prevalent condition among women of reproductive age and has been consistently associated with an increased susceptibility to thyroid dysfunction during pregnancy. This relationship was clearly demonstrated by Männistö et al., who investigated thyroid function in the first trimester of pregnancy in women with adequate iodine intake, negative thyroid autoantibodies, and no prior history of thyroid disease. Their findings revealed a significant correlation between BMI and thyroid hormone levels. Specifically, higher BMI values were associated with increased serum concentrations of TSH and fT3 along with decreased levels of fT4 [87].

Several mechanisms may explain the association between obesity and thyroid dysfunction. First, higher BMI is linked to increased TBG levels, which can lower fT4 and trigger a feedback-driven rise in TSH [88,89]. Second, obese women exhibit a blunted thyroid response to hCG stimulation in pregnancy [90]. Third, the elevated leptin levels characteristic of obesity act as neuroendocrine regulators that correlate with TSH secretion and peripheral T4-to-T3 conversion [91,92]. In addition, leptin exerts significant immunomodulatory effects. Farooqui et al. demonstrated that leptin administration restores T-cell counts and promotes cytokine production, suggesting a biological rationale for its possible involvement in thyroid autoimmunity [93]. Consistent with this, Marzullo et al. observed a higher prevalence of hypothyroidism and autoimmune thyroid disease in obese individuals and correlation analysis showed that leptin levels were associated with thyroid autoimmunity independent of bioanthropometric variables [94]. However, it is important to emphasize that these findings establish a clinical association rather than a direct causal relationship, and the mechanistic role of leptin in human gestational thyroid dysfunction remains to be fully elucidated.

Despite these biological links, recent large-scale data emphasize that BMI’s independent predictive power for gestational thyroid dysfunction remains modest. For instance, recent evidence shows that BMI is associated with a modest risk of having a TSH > 4 mU/L, independent of autoimmunity or familiar predisposition (OR 1.125; 95%CI: 1.08–1.17) [95]. Also, Osinga et al. have shown that a higher BMI was associated with a slightly higher risk of thyroid dysfunction. In particular, for BMI from 20 to 40 kg/m^2^, the risk of overt hypothyroidism increased from 0.5% to 0.8% (OR 1.03; 95%CI: 1.01–1.06), while the risk of subclinical hypothyroidism did not change (OR 1; 95%CI: 0.99, 1.01). Consequently, even though obesity influences the thyroid axis, it appears to be a secondary risk factor compared to autoimmunity and iodine status.

### 3.5. Ethnicity

Ethnicity has emerged as a potential risk factor for thyroid dysfunction during pregnancy, contributing to a growing emphasis on personalized diagnostic approaches. A few studies have documented significant ethnic differences in maternal thyroid parameters and in the prevalence of thyroid autoimmunity during both pregnancy and the postpartum period. When comparing the thyroid function of African-American and Caucasian women during gestation and the postpartum period, Walker et al. found that African-American women had significantly lower TSH levels at all time-points [96]. According to the authors, the lower TSH levels in African-American women were primarily due to higher hCG compared to Caucasian women rather than thyroid autoimmunity or iodine status. However, hCG did not fully explain the TSH variations across different groups [96]. Consistent with these data, a cross-sectional study on 1683 pregnant women showed that prevalence of subclinical hypothyroidism was higher in women of Caucasian background as compared to sub-Saharan and North African backgrounds [97]. These findings were further validated by a large-scale analysis of 9767 women in early pregnancy from the Generation R and ABCD cohorts. The study identified ethnicity as a significant—albeit modest—determinant of maternal TSH: compared to Caucasian women, the risk of elevated TSH was significantly lower in Moroccan (OR 0.40; 95%CI: 0.22, 0.76) and Surinamese women (OR 0.38; 95%CI: 0.21, 0.69). Conversely, no significant differences were observed for Turkish, Asian, or other non-Western backgrounds, highlighting the complexity of ethnic-specific variations in thyroid function [98]. Theoretically, these variations may stem not only from hCG but also from genetic differences in thyroid hormone pathways, as genetic factors account for approximately 40-65% of inter-individual TSH variability [34].

### 3.6. Human Chorionic Gonadotropin

Human chorionic gonadotropin (hCG) is a pregnancy-specific hormone secreted by the syncytiotrophoblast, whose primary functions include the maintenance of the corpus luteum to sustain progesterone production and the suppression of the maternal immune response [99]. It becomes detectable in maternal serum as early as eight days after fertilization, and its levels peak between the 8th and 10th week of gestation, followed by a gradual decline that continues until term [99,100]. The structural homology between hCG and TSH explains hCG’s weak agonistic activity at the TSH receptor (TSH-R), which leads to increased thyroid hormone production during gestation and a reciprocal suppression of serum TSH [101]. Indeed, a TSH level below 0.4 mU/L is observed in up to 15% of healthy women during the first trimester [1]. This phenomenon, resulting in gestational transient thyrotoxicosis (GTT), affects approximately 5% of European pregnancies [102,103], though prevalence varies significantly with ethnicity, reaching 11% in Asian populations during the first trimester [104] and 15% in Pacific Islanders [103]. Notably, the fraction of women with a suppressed TSH typically decreases as pregnancy progresses, falling to about 10% in the second trimester and 5% in the third trimester [1].

While the effect of hCG is usually subclinical, overt thyrotoxicosis can develop under conditions with markedly elevated hCG, such as hydatidiform mole or severe hyperemesis gravidarum [101,105]. Also, TSH-R polymorphisms that enhance the receptor sensitivity to hCG may lead to severe forms of thyrotoxicosis [106,107,108]. This was described for the first time in 1998 by Rodien et al., who identified a heterozygous missense mutation at position 183 of the TSH-R, resulting in the substitution of lysine with arginine and an increase in receptor sensitivity [106]. This condition required treatment with antithyroid medication and resolved spontaneously following delivery [106]. Similar variants, such as the substitution of lysine with asparagine at position 183 or the V597I mutation in the transmembrane domain, have since been confirmed as causes of recurrent gestational thyrotoxicosis [107,108].

In addition, hCG levels are significantly higher in multiple pregnancies, which carries a substantially increased risk of TSH suppression and gestational transient thyrotoxicosis compared to singleton gestations. A recent systematic review by Liu et al. shows that higher hCG levels in multiple pregnancies carry a significantly higher risk of GTT, as the absolute risks for GTT were 3–7.2% and 1.2–2.2% in cases of twin pregnancy and singleton pregnancy [109]. By contrast, there was no convincing overall association of twin pregnancy with subclinical hypothyroidism [109].

Recent evidence suggests that the impact of hCG extends across the entire spectrum of thyroid function. As demonstrated by Korevaar et al. [90], relatively low hCG levels or a suboptimal thyroidal response to hCG stimulation correlate with an increased risk of maternal hypothyroxinemia. This blunted response is particularly evident in TPOAb-positive women, where autoimmunity decreases the functional capacity of the gland [110,111], and has been associated with adverse outcomes such as premature delivery [112] and reduced fetal growth [113].

Last, it is worth mentioning that controlled ovarian hyperstimulation (COH) for in vitro fertilization (IVF) significantly alters hormonal balance due to the administration of hCG for oocyte maturation as well as high estradiol levels that increase TBG and reduce free thyroid hormones [114]. IVF has been associated with a risk of hypothyroidism and TSH seems to increase by a mean of 0.69 mU/L during COH [114]. However, large-scale data from Osinga et al. found no significant association between IVF and overt hypothyroidism (OR 1.90; 95%CI 0.69, 5.23) [36].

### 3.7. Placental Angiogenic Factors

During pregnancy, there is an increase in placental angiogenic factors, such as the pro-angiogenic placental growth factor (PlGF) and the anti-angiogenic soluble fms-like tyrosine kinase 1 (sFlt-1). In 2004, Levine et al. reported the results of a nested case-control study demonstrating the presence of increased levels of sFlt-1/PlGF in women with pre-eclampsia [115]. These changes were detected not only once disease was clinically apparent, but also several weeks earlier, such that the sFlt-1/PlGF ratio was proposed as a predictor of development of pre-eclampsia [115]. Based on these data, pre-eclampsia is now regarded as a condition arising from an imbalance between angiogenic factors, where high levels of sFlt-1 lead to placental vascular insufficiency and other systemic manifestations of pre-eclampsia by antagonizing the angiogenic and vasodilatory effects of PlGF [116].

Recent data suggest that these angiogenic factors might influence the thyroid function of pregnant women as well. In particular, animal studies suggest that these factors might reduce thyroid vascular density, causing an increase in TSH or a decrease in fT4 [117]. In humans, in newborn umbilical cord blood, sFlt-1 was associated with an increase in TSH and a decrease in fT4, while PlGF was associated with an increase in fT4 [118]. Consistent with this, high maternal levels of sFlt-1 have been associated with decreased fT4 [119]. Interestingly, in a recent meta-analysis of individual-participant data, maternal subclinical hypothyroidism was associated with a higher risk of pre-eclampsia [120]. Although the mechanism underlying this association could be the relative deficiency in thyroid hormones, which would promote endothelial cell dysfunction, leading to pregnancy-induced hypertension, the authors argued that the association between subclinical hypothyroidism and pre-eclampsia could be due to reverse causation [120]. In other words, rather than subclinical hypothyroidism increasing the risk of pre-eclampsia, it could be the anti-angiogenic profile that arises in the early stages of pre-eclampsia that might adversely affect thyroid gland vascularization. This hypothesis is in line with the results of another study by Levine et al., showing that in women with pre-eclampsia as well as in normotensive controls, the extent of increase in TSH during pregnancy was directly related to the magnitude of circulating sFlt-1 before delivery [121]. In addition, the authors found that women with a history of pre-eclampsia in the first and second pregnancies were more likely than other women to have non-autoimmune subclinical hypothyroidism over the years. This was seen in women with pre-eclampsia in one pregnancy (OR 2.6; 95%CI: 1.3, 5.0), and it was particularly strong in those who had experienced pre-eclampsia in two pregnancies (OR 5.8; 95%CI: 1.3, 25.5) [121].

## 4. Clinical Utility of Refining Risk-Based Screening: Evidence and Controversies

All these data lead to the question as to whether we can improve targeted thyroid screening. Today, major scientific societies like the ATA still lean towards targeted thyroid screening due to the lack of definitive evidence from large clinical trials on the benefits of treating subclinical hypothyroidism in all pregnant women. As long as the screening strategy remains controversial, refining the predictive power of clinical risk factors is a necessary and pragmatic step to ensure high-risk women are not missed.

Consistent with this concept, Sitoris et al. carried out a cross-sectional study on 1663 pregnant women to determine whether the detection rate of subclinical hypothyroidism and overt hypothyroidism could be improved by adding Caucasian background, BMI ≥ 30 kg/m^2^, and iron deficiency to the established ATA risk factors [122]. Thyroid function was tested in all the women (universal screening) and the overall prevalence of increased TSH with normal fT4 was 4.5% for subclinical hypothyroidism and 0.5% for overt hypothyroidism. The authors demonstrated that if thyroid function was tested based strictly on ATA guidelines, only 1068/1663 women would be evaluated and the detection rate of subclinical hypothyroidism would be only 2.4%, effectively missing nearly half of the cases identified by universal screening. If the screening criteria were expanded to include ethnic background and BMI ≥ 30 kg/m^2^, 1265/1663 women would be identified and the detection rate of subclinical hypothyroidism would rise to 3.7%, a figure comparable to that of universal screening. Furthermore, adding iron deficiency to these criteria increased the evaluation to 1389/1663 women, achieving a detection rate of 4%. The authors concluded that adding Caucasian background and BMI ≥ 30 kg/m^2^ improves case finding and proposed adding them to the list of ATA risk factors, as they are easy to verify and do not add costs to screening [122].

Nevertheless, more recently, a large individual-participant data meta-analysis aiming to re-evaluate the predictive strength of currently accepted risk factors for thyroid dysfunction during pregnancy questioned their clinical utility for targeted screening. Osinga et al. found that maternal age, BMI, smoking status, parity, and gestational age had poor predictive ability for detecting overt or subclinical hypothyroidism, [36]. Crucially, only TPOAb positivity was a relevant risk factor for subclinical hypothyroidism (OR 8.42; 95% CI 7.60–9.62) [36].

Recently, Liu et al. (2025) conducted a comprehensive systematic review to validate established risk factors and explore potential novel indicators [109]. The authors evaluated a wide range of variables, including biochemical markers (hCG, iron, iodine, sFlt-1, and PlGF) environmental exposure to EDCs, as well as obstetric and reproductive history (parity, gravidity, IVF, twin pregnancy, history of infertility, pregnancy loss, preterm delivery, abnormal uterine bleeding, or pre-eclampsia). Table 1 presents the odds ratios for overt and subclinical hypothyroidism for the risk factors included in the ATA and the RCOG guidelines, based on the quantitative data synthesized by Liu et al. Consistent with the results of Osinga, the authors concluded that, among the many candidate factors, only thyroid antibody positivity remains the most relevant risk factor for both overt and subclinical hypothyroidism. In particular, they observed that while factors such as BMI and age show statistical associations, they contribute only marginally to the absolute risk. For most other factors recommended in current guidelines, the supporting evidence was weak, mixed, or insufficient [109].

In conclusion, these data underscore that while expanding targeted screening criteria to include factors such as obesity, ethnicity, or iron deficiency may improve case detection, thyroid autoimmunity, and moderate-to-severe iodine deficiency remain the most consistent and reproducible predictors of thyroid dysfunction. Both conditions are robustly associated not only with subclinical hypothyroidism but also with significant pregnancy complications, including pre-eclampsia, preterm birth, and impaired neurocognitive development in offspring. Consequently, clinicians should ensure optimal iodine intake starting from the preconception period and might consider incorporating both TPOAb and TgAb assessment into risk-based screening strategies.

## 5. Conclusions

Guidelines recommend risk-based screening for thyroid dysfunction in pregnancy. Improving the predictive power of this approach is not merely an academic exercise. It is a necessary step to ensure that high-risk women are not missed while awaiting more robust data from prospective randomized trials on the absolute cost-benefit ratio of universal screening. Established risk factors, such as personal history, autoimmunity, and iodine deficiency, are now joined by emerging risk factors including iron deficiency, EDCs, BMI, ethnicity, and placental-related factors. Unfortunately, recent large-scale data have failed to demonstrate a strong predictive ability of the majority of these for detecting overt or subclinical hypothyroidism, questioning their clinical utility for targeted screening. To date, while the debate regarding the optimal screening strategy persists, the most consistent and reproducible predictors of thyroid dysfunction in pregnancy remain thyroid autoimmunity and moderate-to-severe iodine deficiency.

## Figures and Tables

**Table 1:** ATA and ROCG risk factors that should trigger thyroid function testing, along with odd ratios [109].

ATA 2017	RCOG 2025	Effect Estimate (95% CI)
**General characteristics**		
Age > 30 years	Not included	OH: OR 1.02 (1.00, 1.05) [36] SH: OR 1.02 (1.01, 1.03) [36]
BMI ≥ 40 Kg/m^2^	Not included	OH OR 1.03 (1.01, 1.06) [36] SH: OR 1.00 (0.99, 1.01) [36]
**History presence suspicion of a thyroid disease**		
History or signs and symptoms of thyroid dysfunction	Previous thyroid dysfunction or thyroiditis or discriminatory signs and symptoms (cardiac dysrhythmias; significant preconception weight loss; enlarging thyroid gland)	OH: RR 0.40 (0.05; 3.10) [123] SH: RR 0.36 (0.13, 0.98) [123]
Presence of a goiter	Previous thyroid surgery/goiter/nodules	Not found
**Thyroid autoimmunity or autoimmune conditions**		
Thyroid antibody positivity	Known TPOAb positivity	OH: OR 24.2 (18.5, 31.6) [36] SH: OR 8.42 (7.60, 9.32) [36]
T1DM or other autoimmune disease	T1DM; SLE; Anti-Ro/Anti-La positivity; Anti-phospholipid syndrme	OH: not found SH: RR 4.8 (1.3, 18.2) [124]
Family history of thyroid autoimmune disease or dysfunction	Not included	OH not found SH: OR 2.88 (0.65, 12.9) [125] SH: RR 3.4 (1.8, 6.2) [124]
**Exposure to risk factors for thyroid disease**		
Residing in an area of moderate-to-severe iodine deficiency	Not included	OH: OR 9.15 (2.78; 30.2) [126] SH: OR 2.47 (1.22, 5.71) [48]
History of head/neck radiation or prior thyroid surgery	Previous head/neck irradiation	
Use of amiodarone, lithium, administration of contrast media	Previous RAI; recent current thyroid disruptive medication (amiodarone/lithium)	
**Obstetric history**		
History of pregnancy loss, preterm delivery, or infertility	Stillbirth; second trimester miscarriage	OH: not found SH: OR 2.75 (0.80, 9.48) [125] ^a^ SH: OR 2.44 (0.98, 6.08) [125] ^b^
Multiple prior pregnancies (≥2)	Not included	OH: OR 0.88 (0.54, 1.37) [36] SH: OR 0.94 (0.80, 1.11) [36]

^a^ Pregnancy loss. ^b^ Infertility. TPOAb, thyroid peroxidase antibody; BMI, body mass index; NA, not applicable; OH, overt hypothyroidism; OR, odds ratio; RAI, radioiodine ablation; RR, rate ratio; SH, subclinical hypothyroidism; SLE, systemic lupus erythematosus; type 1 diabetes mellitus.

## Data Availability

Not applicable.
